# Urinary Retinol Binding Protein Is a Marker of the Extent of Interstitial Kidney Fibrosis

**DOI:** 10.1371/journal.pone.0084708

**Published:** 2014-01-08

**Authors:** Nicolas Pallet, Sophie Chauvet, Jean-François Chassé, Marc Vincent, Paul Avillach, Charlene Levi, Vannary Meas-Yedid, Jean-Christophe Olivo-Marin, Diane Nga-Matsogo, Philippe Beaune, Eric Thervet, Alexandre Karras

**Affiliations:** 1 INSERM U775, Centre Universitaire des Saints Pères, Paris, France; 2 Service de Biochimie, Hôpital Européen Georges Pompidou, Assistance Publique-Hôpitaux de Paris, Paris, France; 3 Service de Néphrologie, Hôpital Européen Georges Pompidou, Assistance Publique-Hôpitaux de Paris, Paris, France; 4 Unité d’Analyse d’image quantitative, Institut Pasteur, Paris, France; 5 Departement d’informatique médicale, Hôpital Européen Georges Pompidou, Assistance Publique-Hôpitaux de Paris, Paris, France; 6 Université Paris Descartes, Paris, France; Biomedical Research Foundation of the Academy of Athens, Greece

## Abstract

Currently, a non-invasive method to estimate the degree of interstitial fibrosis (IF) in chronic kidney disease is not available in routine. The aim of our study was to evaluate the diagnostic performance of the measurement of urinary low molecular weight (LMW) protein concentrations as a method to determine the extent of IF. The urines specimen from 162 consecutive patients who underwent renal biopsy were used in the analysis. Numerical quantification software based on the colorimetric analysis of fibrous areas was used to assess the percentage IF. Total proteinuria, albuminuria, and the urinary levels of retinol binding protein (RBP), alpha1-microglobulin (α1MG), beta 2-microglobulin (β2MG), transferrin, and IgG immunoglobulins were measured. There was a significant correlation between the degree of IF and the RBP/creatinine (creat) ratio (R2: 0.11, p<0.0001). IF was associated to a lesser extent with urinary β2MG and α1MG; however, there was no association with total proteinuria or high molecular weight (HMW) proteinuria. The correlation between IF and RBP/creat remained significant after adjustment to the estimated glomerular filtration rate, age, body mass index, α1MG, and β2MG. The specificity of the test for diagnosing a fibrosis score of >25% of the parenchyma was 95% when using a threshold of 20 mg/g creat. In conclusion, RBP appears to be a quantitative and non-invasive marker for the independent prediction of the extent of kidney IF. Because methods for the measurement of urinary RBP are available in most clinical chemistry departments, RBP measurement is appealing for implementation in the routine care of patients with chronic kidney disease.

## Introduction

Kidney disease evolution invariably lead to interstitial fibrosis (IF), which is the main factor contributing to kidney structural deterioration and loss of function [1], [2], and its extent is positively correlated with adverse outcomes [3], [4], [5], [6]. Therefore, IF may be may be considered a surrogate marker and its evaluation is of important prognostic value

The development of non-invasive biomarkers of kidney status and outcome is challenging but can result in individualized therapies for patients with chronic kidney disease (CKD) [7], [8]. Although experimental and clinical evidence has contributed greatly to our knowledge of the pathophysiological role of the various molecular components of proteinuria in the progression of chronic kidney injury, namely, inflammation, tubular atrophy (TA), and IF, none of the urinary markers used routinely in clinical chemistry has been investigated as a predictor of the extent of IF [9], [10]. Regardless, current evidence indicates that low molecular weight (LMW) and/or high molecular weight (HMW) proteinuria could serve as a non-invasive marker of kidney histological damage and may predict kidney survival in specific patients, including kidney transplant recipients (KTRs) and CKD patients [11], [12], [13]

In this study, we have hypothesized that IF (independent of the cause) promotes a defect in the proximal reabsorption of LMW proteins and that the urinary concentration of these proteins is an indication of the extent of kidney fibrosis. To test this hypothesis, we measured the urinary concentration of LMW proteins (retinol-binding protein, RBP; β2-microglobulin, β2MG; and α1-microglobulin, α1MG), HMW proteins (albumin, Alb; transferrin, TRF; and immunoglobulins G, IgG), and total proteins in 189 consecutive patients who underwent a kidney biopsy. Next, we measured the strength of the association between these proteins and the percentage IF, measured using a color segmentation image analysis technique. Our data indicated that the urinary concentration of LMW proteins was positively correlated with the degree of IF, and urinary RBP was independently associated with the extent of IF in CKD patients.

## Methods

### Patient population

Between March 2012 and March 2013, 189 consecutive patients who were referred to the Nephrology Department at the Georges Pompidou European Hospital (Paris, France) for kidney biopsy were evaluated for potential inclusion in the study. Indications for biopsy were estimated GFR<60 ml/min and/or proteinuria>0.5 g/l. Kidney biopsies were not performed for the purpose of this non interventional study, but only for patient care. We excluded from the study all patients with acute kidney injury and KTRs. At the time of biopsy, urine and blood samples were collected for routine clinical chemistry analyses and stored at −80°C. Detailed information regarding the clinical, medical, demographic, biological, and histological status of the patients was collected using an information-based data warehouse [14]. Analysis were performed anonymously. Paris Descartes University ethics comity (Comité de Protection des Personnes/Patients Protection Comity, representative: Dr Marie-France Mamzer) approved this observational study. Research was not performed out of France. Participants provided written consents, and the ethic committee approved this consent procedure. There is no difference in the distribution of histological diagnoses compared with the previous year (2011–2012), which confirms that this study is observational. The prevalence of glomerular diseases was 55% in 2012–2013 compared with 51% in 2011–2012; the prevalence of tubular and interstitial diseases was 22% in 2012–2013 compared with 25% in 2011–2012; the prevalence of vascular diseases was 16% in 2012–2013 compared with 15% in 2011–2012.

### Clinical chemistry analyses

The urine protein measurements were performed at the Clinical Chemistry Department of the European Georges Pompidou Hospital. The urinary total protein concentration was quantified by measuring pyrogallol red at 600/800 nm absorbance (urinary CSF protein assay, Beckman Coulter), and the Alb concentration was measured by immunoturbidimetry analysis (DIAgAM assay, Beckman Coulter) using a Beckman Coulter AU680 analyzer. The urinary levels of α1MG, β2MG, TRF, RBP, and IgG were measured using a Siemens BN II nephelometer Analyzer II and kits from Siemens. Values considered normal were as follows: α1MG<15 mg/L; β2MG<350 μg/L; RBP<0.5 mg/L; TRF<2.2 mg/L, IgG<3.6 g/L, and Alb<20 mg/L [15], [16]. The urine samples were alkalinized prior to measuring the β2MG level. The urine protein concentrations were corrected for creatinine levels, which were measured using a colorimetric assay (modified kinetic Jaffe method) on a Beckman Coulter DXC analyzer (serum) or a Beckman Coulter AU680 analyzer (urine). The glomerular filtration rate (eGFR) was estimated using the modification of the diet in renal disease (MDRD) formula [17].

### Automated measurement of interstitial fibrosis

Masson's trichrome-stained kidney sections were analyzed using color segmentation image analysis software for the quantification of IF [18]. An image of the cortical section of each kidney biopsy was captured using the Hamamatsu slide scanner, Nanozoomer with an objective 20×, NA = 0.75, and a Hamamatsu 3-CCD camera. The cortex areas of the biopsy were analyzed, whereas the medulla was excluded from analysis. The images were analyzed using color segmentation image analysis software that automatically extracts the green color areas characteristic of IF; the renal capsule, tubular basement membranes, glomeruli, and vessel were automatically excluded from the analysis. The method for fibrosis quantification has been developed in details elsewhere. Briefly, the protocol for quantification consists of two steps: (1) segmentation to extract green areas and the biopsy area; (2) postprocessing to compute the IF index. The postprocess to compute the IF index is based on the exclusion of non-fibrotic areas. In order to keep only the IF area, the following regions are automatically detected and excluded from analysis. The index of the IF surface is defined as the ratio between the IF surface area and the total surface of the cortex area in the biopsy. More technical details can be found in [18] and particularly in the supplemental part (http://links.lww.com/TP/A493).

### Statistical analysis

The results were expressed as the frequencies and percentages for the categorical variables and as the mean ± SEM for the continuous variables. Associations between the clinical and biological features of the patients were tested using the Student T test, Mann-Whitney test or through the estimation of the Pearson correlation coefficient (Pearson coefficient  = √R^2^), where appropriate. The variables correlating with the extent of IF by univariate analysis were included in a multivariate linear regression model. The variables correlating with the extent of IF by univariate analysis were included in a multivariate linear regression model. To check that despite this overlap RBP can help improve the prediction of fibrosis, we fitted and compared two linear models, one including known predicting factors alone (α1MG, β2MG and DFG) and one including both these factors and RBP. The two models were compared based on a Likelihood ratio test, where the deviance follows a Chi-square distribution with number of degrees equal to the difference of included variables in the two nested models. A backward stepwise linear regression analysis was used to identify the clinical factors independently associated with the extent of the IF. Power calculation is not necessary, because our principal objective was not to compare two groups of patients towards an end point to test the null hypothesis, with an alpha and beta risk. Rather, we performed uni and multivariate linear regression analysis; and we evaluated the performance of a diagnostic biomarker. The statistical analyses were performed using the R framework (http://www.R-project.org/).

## Results

### Characteristics of CKD patients

**Figure 1:**
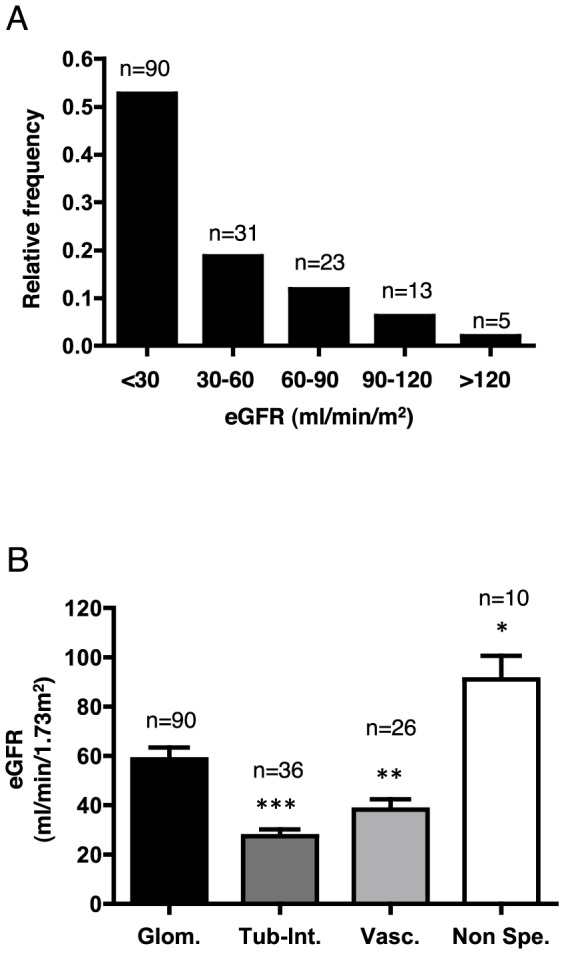
Distribution of estimated glomerular filtration rates. **A**. Histogram showing the distribution of the study population according to the eGFR level at inclusion. **B**. Histogram showing eGFR values according to the type of kidney disease. eGFR is expressed as the mean ± sem. *p<0.05, **p<0.01, ***p<0.001 compared to glomerular disease using Student's T test; the Mann-Whitney test was used for the glomerular vs. comparison with non-specific lesions. Glom: glomerular diseases; Tub-Int: tubular and/or interstitial diseases; Vasc: vascular diseases; Non-spe: non-specific.

Of the 189 patients who entered the study between March 2012 and March 2013, 27 were excluded because of the absence of an analyzable histological sample (less than 10 glomeruli). The demographic and medical characteristics of the remaining 162 patients are listed in **Table 1** **.** Importantly, approximately half of the patient population was not of Caucasian origin. The vast majority of the kidney disease etiologies were of glomerular origin, which may explain the high mean level of proteinuria (2.3 g/L). Advanced kidney disease was common; more than 50% of the patients exhibited an eGFR <30 ml/min (**Fig. 1A**). The patients with glomerular disease exhibited better renal function compared to those with tubulo-intertitial or vascular nephropathy (**Fig. 1B**).

**Table 1:** Baseline characteristics of the study cohort (n = 162).

**Demographics**	
Age (years)	52.6±18.2
Gender (male)	88 (54)
Caucasian patients	86 (53)
Body Mass Index (kg/m^2^)	25.4±4.8
Hypertension	79 (48)
Hyperlipidemia*	37 (22)
Diabetes	17 (10)
Cancer**	25 (15)
**Etiology of CKD**	
FSGS/minimal changes disease	23 (14)
ANCA-mediated vasculitidis	15 (9)
IgA nephropathy	14 (8.5)
Primary Glomerulonephritis	14 (8.5)
Lupus	11 (6.5)
Diabetes	10 (6)
Tubule-interstitial nephropathy	10 (6)
Hypertensive nephropathy	10 (6)
Non specific***	10 (6)
Myeloma cast nephropathy	9 (5.5)
Other	36 (22)
**Medications**	
ACEI/ARB	69 (43)
Diuretics	27 (17)
Beta blockers	19 (12)
Calcium Channel Inhibitors	37 (23)
Others	7 (4)
**Clinical Chemistry**	
Serum creatinine (mg/dl)	2.08±1.45
eGFR (ml/min/1.73 m^2^)	47.4±33.3
Proteinuria (g/l)	2.3±2.7
Albuminuria (g/l)	1.3±2.4

Continuous variables are expressed as the mean ± sem; categorical variables are expressed as n (%).

Hyperlipidemia: total cholesterol >200 mg/dl and/orTriglycerides >200 mg/dl, and/or LDL cholesterol>130 mg/dl.

Cancer: Multiple myemoma ( = 13), Urogenital ( = 7), Breast ( = 2), Lung ( = 1).

Non specific: no diagnosis, moderate interstitial fibrosis and/or glomerulosclerosis.

### LMW proteinuria but not HMW proteinuria is associated with low eGFR

**Figure 2:**
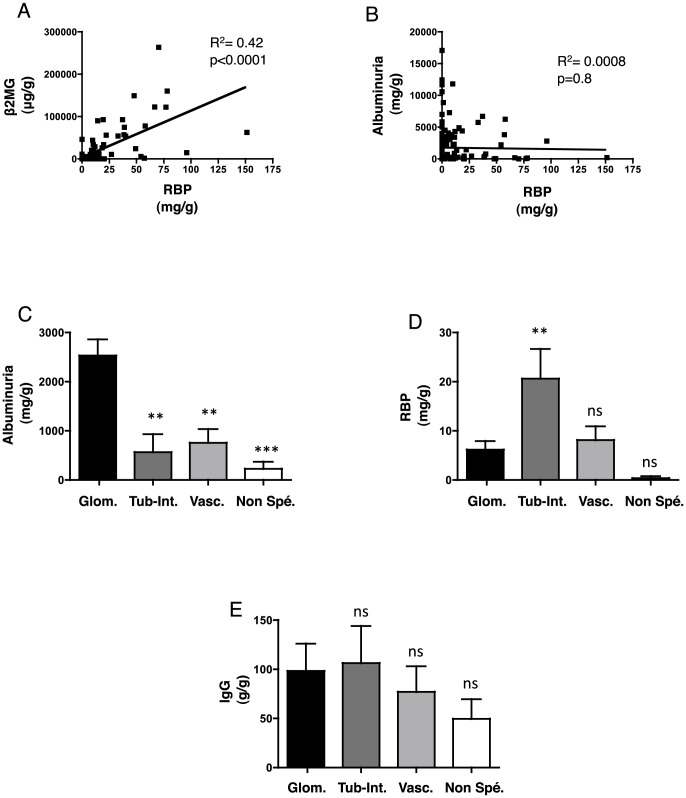
Expression patterns of proteinuria. **A**, **B**. Best-fit slope of the linear regression between RBP and β2MG (A) and RBP and Alb (B). **C**, **D**, **E**. Histogram showing Alb (A), RBP (B), and IgG (C) values according to the type of kidney disease. Protein concentrations are expressed as the mean ± sem. **p<0.01, ***p<0.001 compared to glomerular disease using Student's T test; the Mann-Whitney test was used for the glomerular vs. comparison with non-specific lesions. Glom: glomerular diseases; Tub-Int: tubular and/or interstitial diseases; Vasc: vascular diseases; Non-spe: non-specific.

We characterized the proteinuria expression patterns in the 162 CKD patients. The LMW proteins strongly correlated with each other but not with the HMW proteins and total proteinuria (**Fig. 2A and B** **,** **Table 2** **, Fig. S1A-C**). As expected, HMW proteinuria was significantly higher in patients with glomerular disease compared to patients with tubular and vascular diseases (**Fig. 2C**, **Fig. S1D**), whereas LMW proteinuria was higher in patients with tubular disease compared to patients with vascular and glomerular diseases (**Fig. 2D**, **Fig. S1E and F**). Although a higher IgG concentration was expected in patients with glomerular disease (**Fig. 2E**), IgG concentration surprisingly did not discriminate a particular group of diseases. Total proteinuria was only weakly higher in the patients with glomerular disease than in patients with the other diseases (p = 0.046) (**Fig. S1G**).

**Table 2:** Summary of correlative data.

Covariate	R^2^	p
**Retinol Binding Protein**		
β2MG	0.42	<0.0001
α1MG	0.66	<0.0001
Proteinuria	0.02	0.07
TRF	0.006	0.3
Albuminuria	0.0008	0.8
Fibrosis (glomerular diseases)	0.18	0.001
Fibrosis (tubule and interstitial diseases)	0.1	0.2
Fibrosis (vascular diseases)	0.008	0.5
**eGFR**		
Fibrosis (glomerular)	0.26	<0.0001
Fibrosis (vascular)	0.05	0.2
Fibrosis (tubule and interstitial diseases)	0.001	0.5
**Fibrosis**		
RBP	0.11	<0.0001
α1MG	0.08	0.0004
β2MG	0.03	0.03
TRF	0.01	0.2
Proteinuria	0.007	0.2
IgG	0.002	0.5
Albuminuria	0.0002	0.8

**Figure 3:**
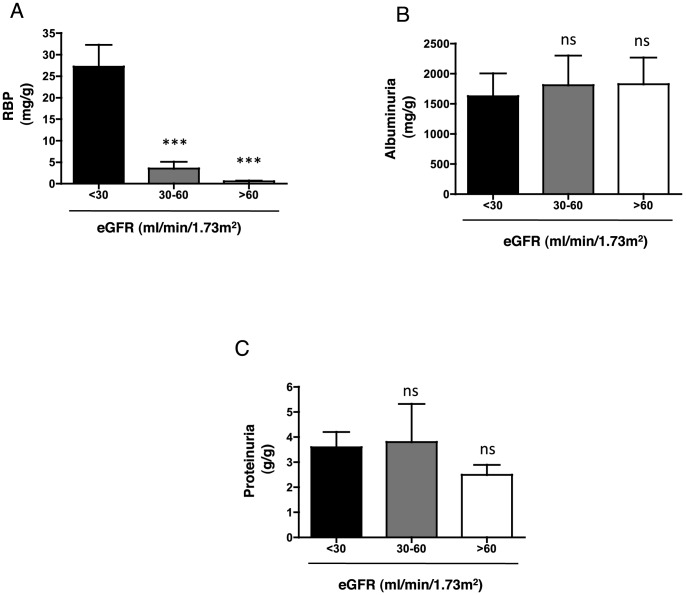
Distribution of proteinuria according to the estimated glomerular filtration rate. **A**, **B**, **C**. Histogram showing Alb (A), RBP (B), and total proteinuria (C) values according to the level of eGFR. Protein concentrations are expressed as the mean ± sem. **p<0.01, ***p<0.001 compared to eGFR <30 ml/min, using Student's T test.

The distribution of proteinuria according to the eGFR levels was analyzed. LMW proteinuria was significantly higher among the patients with a mean eGFR value <30 ml/min (**Fig. 3A**, **Fig. S2A and B**) compared to the patients with eGFR values >30 ml/min, whereas HMW and total proteinuria were not significantly different among the eGFR groups (**Fig. 3B and C**, **Fig. S2C and D**). These data indicated that LMW proteinuria but not total or HMW proteinuria was a characteristic of patients with low eGFR in this CKD population.

### The extent of IF is correlated with a lower eGFR

**Figure 4:**
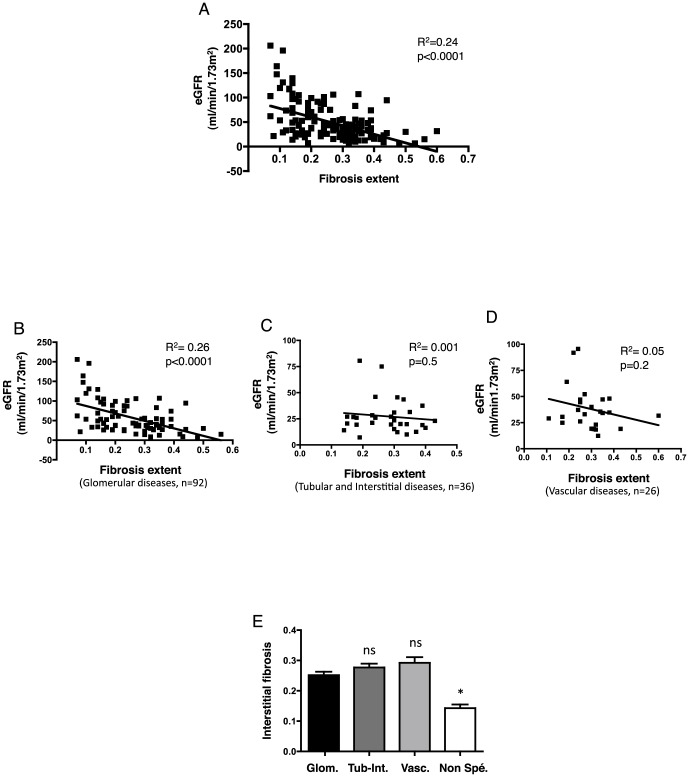
Correlation between the estimated glomerular filtration rate and extent of interstitial fibrosis. **A**. Best-fit slope of the linear regression between eGFR and extent of fibrosis. **B**, **C**, **D**. Best-fit slope of the linear regression between eGFR and extent of fibrosis in glomerular disease (B), tubular disease (C), and vascular disease (D). **E**. Histogram of the distribution of the extent of interstitial fibrosis according to the type of kidney disease. The percentages of interstitial fibrosis are expressed as the mean ± sem. *p<0.05 compared to glomerular disease, using Student's T test; the Mann-Whitney test was used for the glomerular vs. non specific lesions comparison. Glom: glomerular diseases; Tub-Int: tubular and/or interstitial diseases; Vasc: vascular diseases; Non-spe: non-specific.

We next analyzed the characteristic features of IF in the CKD patients. As proof of the deleterious effect of interstitial fibrosis in CKD, we determined that the extent of IF was negatively associated with the eGFR (R^2^ = 0.24, p<0.0001) (**Fig. 4A** **,** **Table 2**), and that this correlation was highly significant for glomerular disease but not for tubular or vascular diseases (**Fig. 4B-D**). There was no difference in the IF percentages between the types of nephropathies even though, as expected, the IF percentage in the normal biopsies was significantly lower (**Fig. 4E**). These results indicated that in our population, the extent of IF was correlated significantly with a low eGFR, at least in patients with glomerular disease.

### LMW proteinuria but not HMW proteinuria is correlated with higher IF

**Figure 5:**
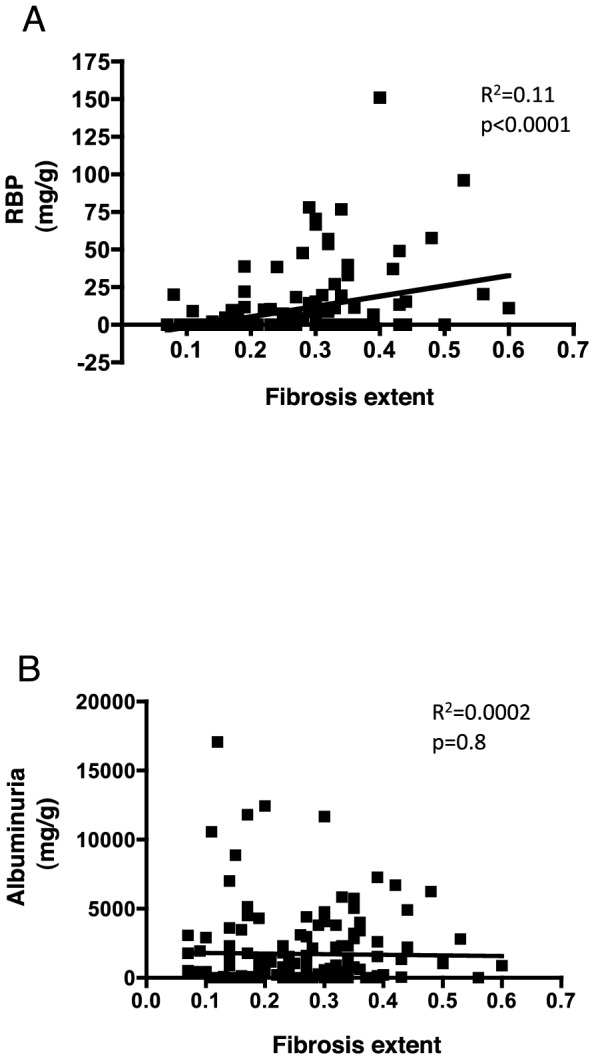
Correlation between proteinuria and the extent of interstitial fibrosis. **A**, **B**. Best-fit slope of the linear regression between RBP (A) and Alb (B) and extent of fibrosis extent.

We next examined the association between LMW proteinuria and the extent of IF. The univariate analysis indicated that LMW proteinuria but not HMW or total proteinuria correlated significantly with the extent of IF; RBP (R^2^ = 0.11, p<0.0001); α1MG (R^2^ = 0.08, p = 0.0004); and β2MG (R^2^ = 0.03, p = 0.02) (**Fig. 5** **,** **Table 2**, **Fig. S3**). This association was significant in patients with glomerular disease (R^2^ = 0.11, p = 0.001) but not in patients with tubule-interstitial disease (R^2^ = 0.10, p = 0.06) or vascular disease (R^2^ = 0.08, p = 0.1) despite a clear trend (**Fig. S4**).

Of the LMW proteins, RBP was the better predictor of IF; therefore, we performed a multivariate analysis to test whether this association was independent of other parameters. Given that α1MG, β2MG, and eGFR were correlated with the extent of IF, indicating that a portion of the information generated by RBP may also be provided by α1MG, β2MG, and eGFR, we fitted and compared two linear models, one including α1MG, β2MG, and eGFR and the other including these factors plus RBP. The two models were compared based on a likelihood ratio test in which the deviance follows a Chi-square distribution with the number of degrees equal to the difference of the variables included in the two nested models. The multiple linear regression analysis indicated that the model with RBP was significantly better than the model without RBP (p = 0.03), demonstrating that RBP was independently correlated with the extent of IF.

Finally, we analyzed the efficiency of testing urinary RBP levels for diagnosing the extent of IF. RBP/creat ratio has a high specificity in detecting cases with >25% fibrosis (the median value of fibrosis in our cohort, which also correlates with an altered prognosis in numerous settings, including chronic allograft nephropathy and chronic kidney diseases [4], [5], [6]; Recipient-Operating Characteristic curve (ROC curve area 0.7, p = 0.0003) indicates a high specificity in detecting cases with >25% fibrosis. For example, an RBP/creat ratio above 20 mg/g predicted a fibrosis score > 25% with a specificity of 95% but with a sensitivity of 20%. The likelihood ratio of this cutoff is 4, which indicates that a patients with a RBP ratio>20 mg/g is 4 times more likely to have >25% fibrosis. This observation indicated that a high concentration of urinary RBP was associated the extent of IF in non-selected CKD patients, with very few false positives; however, a normal or low value did not exclude the presence of IF.

## Discussion

In this study, we demonstrated that the concentration of LMW proteins but not total or HMW proteins was positively associated with the degree of IF and altered renal function. The RBP/creat ratio was independently correlated with the extent of IF and could be useful for predicting renal fibrosis with a high specificity, especially in patients with glomerular diseases. To our knowledge, this study is the first to demonstrate a significant correlation between urinary RBP and kidney IF in a prospective and non-selected population of CKD patients. Given that the extent of fibrosis correlates with adverse outcomes, a non-invasive diagnosis of the extent of interstitial fibrosis is usefull tool for the management of CKD patients, both in terms of prognosis and therapeutic and nephroprotective strategies.

eGFR is a good predictor of fibrosis. The ROC curves of the two predictors are similar in terms of diagnostic performance, and the addition of RBP to eGFR does not increase the performance of the prediction (not shown). This raises the question of the prognostic value of RBP of the decline of renal function, which remains to be examined. Our results suggest that IF promotes loss of function and eGFR decline, and IF promotes tubular dysfunction and RBP loss. This point is supported but the results of the regression analysis indicating that RBP and GFR correlate with IF, and that RBP is independent of GFR.

The proposed rationale for the detection of increased LMW protein concentrations in the urine from patients with extended fibrosis of the kidneys is based on the hypothesis that IF is frequently associated with TA, leading to the defective proximal reabsorption of physiologically filtered LMW. Any increase in the urinary excretion of β2MG or RBP is highly specific for tubular disease, whereas the increased excretion of α1MG is observed in glomerular proteinuria [19], [20]. This non-specific degenerative tubulopathy associated indirectly with LMW output and extended urinalysis (including glucosuria or aminoaciduria) could be used to confirm this hypothesis. There is indirect evidence to support this concept. For example, the reduction in the maximal reabsorption rate of filtered phosphates, which are reabsorbed mainly by the proximal tubules and are excreted in excess urine in cases of proximal tubulopathy, is proportional to the reduction in eGFR [21]. The concept that tubular dysfunction is related to IF is also supported by the recent demonstration of a strong correlation between RBP and Banff cIassification scores and TA in a series of KTRs; the protein measurements correlated with the interstitial Banff scores at the one-year biopsy [22]. However, for reasons that are unclear, the LMW and HMW protein levels observed in the study correlated strongly with each other and with graft function, histology, and survival; associations that were not observed in our study.

An advantage of our study is that the large panel of proteins tested in our center used validated methods used routinely in clinical chemistry laboratories, and the cost was relatively low (7 USD per RBP measurement). In comparison, the cost of a urinary electrophoretic profile is 10.5 USD and an albuminuria measurement is 3 USD. Importantly, the performance of these methods has been validated, including the interlaboratory reproducibility of the tests, and the practical outcome is that almost all clinicians can evaluate the extent of IF in CKD patients through measuring the RBP concentration [23], [24]. To date, few non-invasive markers of the extent of kidney fibrosis have been tested, including polypeptide-like urinary procollagen III aminoterminal propetide (PIIINP) [10]. PIIINP levels have been correlated with the extent of kidney fibrosis; however, the association has not been tested using a multivariate analysis. Moreover, PIIINP analysis is not performed routinely, and the interlaboratory reproducibility of the test is unknown [10]. Regardless, together with our study, these studies demonstrated that non-invasive markers are associated with the extent of IF, which can be evaluated using the robust and reproducible method of color segmentation image analysis. This computerized method for IF quantification has been validated both in KTR [25] and CKD patients [10], and the results are predictive of renal prognosis.

We observed that RBP correlated with IF in sthe group of patients with glomerular disease but not in the patients with tubular or vascular disease. Despite this clear trend, the lack of a positive correlation in these two disease categories may be explained by the relatively low size of these subgroups of patients compared with the glomerular disease subgroup. Additionally, this finding may indicate that the tubule-interstitial injury induced by proteinuria secondary to glomerular disease impedes LMW reabsorption to a greater extent than in primitive tubulo-interstitial diseases. Recently, the effect of LMW proteinuria on the prognosis of glomerular disease was highlighted in a study of membranous nephropathy, demonstrating that LMW proteinuria is associated with the progression of kidney dysfunction[11].

Another intriguing finding was the pattern of urinary IgG expression, which, in contrast to previous studies in KTRs and membranous nephropathies [11], [22], was not associated with any relevant variable in our study. More importantly, the mean concentration of IgG did not differ between etiologic groups, suggesting that despite the high MW of IgG (160 kD), the loss of glomerular permeability does not always result in IgG proteinuria. Our findings are corroborated by the normal IgG excretion rate demonstrated in a series of patients with membranous nephropathy [11]. The possibility of IgG or IgM being produced locally in the interstitium and secreted in the urine has been raised by a number of authors [22]; however, this phenomenon has not been demonstrated. Overall, our study challenged the usefulness of assaying urinary IgG levels in terms of characterizing the type of nephropathy and for purposes of identifying markers of tubulo-interstitial injury.

TA has not been evaluated in this study because to our knowledge, there is no validated automatized method for TA quantification in kidney tissue, and there is a need for tools providing continuous values of TA rather that semi-quantitative score. The only validated system of TA scoring is the Banff system, which is widely used, but with numerous limitations, and provides validated information in kidney transplant patients only. There is always a strong correlation between IF and TA scores in models in which they have be evaluated, mostly in kidney transplant recipients, which indicates the score of IF in somehow reveals the TA. The absence of TA measurement does not change the conclusion of our study regarding the performance of the measurement of the concentrations of RBP in urines in predicting the extent of IF.

In conclusion, we demonstrated that an increase in the excretion of LMW proteins, particularly RBP, is a non-invasive marker that can independently predict the extent of kidney IF. However, further studies are needed to validate our findings. Because the measurement of RBP is implemented routinely in clinical chemistry departments, RBP is an appealing marker to assess the severity of chronic kidney damage. Studies are ongoing to determine whether LMW protein levels independently predict CKD progression.

## Supporting Information

Figure S1 **Distribution of proteinuria in the 162 CKD patients.** A,B,C. Best-fit slope of linear regression between α1MG and RBP (A), TRF and RBP (B), and proteinuria and RBP (C). D, E, F, G. Histograms showing TRF (D), α1MG (E), β2MG (F) and total proteinuria (G) values according to the type of kidney disease. Protein concentrations are expressed as mean±sem. *p<0.05, **p<0.01, ***p<0.001, compared to glomerular disease, Student T test, except Mann-Whitney test for Glomerular vs Non specific comparison and ANOVA for (G). Glom =  glomerular disease; Tub-Int =  tubular and interstitial diseases; Vasc =  vascular diseases; Non-spe =  non-specific lesions.
(EPS)

Figure S2 **Distribution of proteinuria according to estimated glomerular filtration rates.** Histograms showing α1MG (A), β2MG (B), IgG (C) and TRF (D) values according to the type of kidney disease. Protein concentrations expressed as mean±sem. *p<0.05, **p<0.01, ***p<0.001, as compared to eGFR<30 ml/min.
(EPS)

Figure S3 **Correlation between proteinuria and the extent of interstitial fibrosis.** Best-fit slopes of linear regression between α1MG (A), β2MG (B), TRF (C), IgG (D), and proteinuria (E) and the extent of interstitial fibrosis.
(EPS)

Figure S4 **Correlation between proteinuria and the extent of interstitial fibrosis according to the type of nephropathy.** Best-fit slope of linear regression between fibrosis extent and RBP according to the type of nephropathy: glomerular (A), tubular (B), and vascular (C).
(EPS)
